# A phase Ib dose-finding, pharmacokinetic study of the focal adhesion kinase inhibitor GSK2256098 and trametinib in patients with advanced solid tumours

**DOI:** 10.1038/s41416-019-0452-3

**Published:** 2019-04-17

**Authors:** Gabriel Mak, Jean-Charles Soria, Sarah P. Blagden, Ruth Plummer, Ronald A. Fleming, Noelia Nebot, Jianping Zhang, Jolly Mazumdar, Debra Rogan, Anas Gazzah, Ivana Rizzuto, Alastair Greystoke, Li Yan, Jerry Tolson, Kurt R. Auger, Hendrik-Tobias Arkenau

**Affiliations:** 1 Sarah Cannon Research Institute, London, UK; 2 Cancer Centre, University College London, London, UK; 3 Drug Development Department at Gustave Roussy Cancer Campus, University Paris-Sud, Paris, France; 4 Department of Oncology, Imperial College Healthcare NHS Trust, London, UK; 5 Department of Oncology, University of Oxford, Oxford, UK; 6 Northern Institute for Cancer Research, Newcastle University, Newcastle upon Tyne, UK; 7 GlaxoSmithKline, Research Triangle Park, NC and Upper Providence, Collegeville, PA USA; 8 PAREXEL International, Durham, NC USA; 9 Present Address: Novartis Pharmaceuticals Corporation, East Hanover, New Jersey USA; 10 Present Address: Chimeron Bio, New York, NY 10016 USA

**Keywords:** Mesothelioma, Oncology

## Abstract

**Background:**

Combined focal adhesion kinase (FAK) and MEK inhibition may provide greater anticancer effect than FAK monotherapy.

**Methods:**

This dose-finding phase Ib study (adaptive 3 + 3 design) determined the maximum tolerated dose (MTD) of trametinib and the FAK inhibitor GSK2256098 in combination. Eligible patients had mesothelioma or other solid tumours with probable mitogen activated protein kinase pathway activation. Adverse events (AEs), dose-limiting toxicities, disease progression and pharmacokinetics/pharmacodynamics were analysed.

**Results:**

Thirty-four subjects were enrolled. The GSK2256098/trametinib MTDs were 500 mg twice daily (BID)/0.375 mg once daily (QD) (high/low) and 250 mg BID/0.5 mg QD (low/high). The most common AEs were nausea, diarrhoea, decreased appetite, pruritus, fatigue and rash; none were grade 4. Systemic exposure to trametinib increased when co-administered with GSK2256098, versus trametinib monotherapy; GSK2256098 pharmacokinetics were unaffected by concomitant trametinib. Median progression-free survival (PFS) was 11.8 weeks (95% CI: 6.1–24.1) in subjects with mesothelioma and was longer with Merlin-negative versus Merlin-positive tumours (15.0 vs 7.3 weeks).

**Conclusions:**

Trametinib exposure increased when co-administered with GSK2256098, but not *vice versa*. Mesothelioma patients with loss of Merlin had longer PFS than subjects with wild-type, although support for efficacy with this combination was limited. Safety profiles were acceptable up to the MTD.

## Background

Focal adhesion kinase (FAK) is a non-receptor tyrosine kinase whose activity is critical for cancer cell proliferation, survival, migration, and invasion.^1^ FAK overexpression has been documented in several solid tumours, including mesothelioma, and haematological malignancies,^1–5^ but FAK is detected at low levels in normal tissues or benign tumours.^2^ FAK overexpression may relate to prognosis in various human malignancies^3,5^ and is therefore an attractive target for anticancer therapy.

GSK2256098 is a potent, oral, reversible inhibitor of the tyrosine kinase activity measured by the autophosphorylation site (Tyr 397) of FAK.^6^ In preclinical studies, GSK2256098 was demonstrated to be at least 20 times more active *in vitro* in Merlin-negative (encoded by *NF-2*) mesothelioma cells than in Merlin-positive cells.^6^ In a recent phase I study of GSK2256098 monotherapy for patients with advanced solid tumours, mesothelioma patients with Merlin-negative tumours had a progression-free survival (PFS) nearly twice that of Merlin-positive mesothelioma patients.^7^

Trametinib is an oral, small molecule inhibitor of MEK1/2.^8^ MEK1/2 is an enzyme within the mitogen activated protein kinase (MAPK) or RAS/RAF/MEK/ERK pathway. Activation of ERK is commonly observed in cancers, such as mesothelioma, where it occurs in 75% of patients.^9–11^ We hypothesised that a combination of FAK and MEK inhibition may provide greater anticancer effect than FAK monotherapy, supported by preclinical evidence of synergistic growth inhibition and cell death between GSK2256098 and trametinib across a range of mesothelioma cell lines (GSK Internal data; manuscript in preparation).

This study aimed to identify the maximum tolerated doses (MTD) of GSK2256098 and trametinib when administered in combination and to examine the safety, pharmacokinetics, pharmacodynamics, and clinical activity of this combination in patients with mesothelioma or other solid tumours.

## Methods

### Study design

This multipart phase I study (NCT01938443) was conducted at three centres in the UK, and one in France between 7 November 2013 and 24 June 2016.

Part 1 aimed to identify a low GSK2256098/high trametinib and a high GSK2256098/low trametinib MTD, using a standard adaptive 3 + 3 design. Starting doses were GSK2256098 500 mg orally twice daily (BID), 50% of the single-agent MTD, and trametinib 1 mg orally once daily (QD), 50% of the recommended monotherapy dose.^7,12^ Dose de-escalation was followed if the initial doses exceeded the MTD exposure. GSK2256098 was administered with a light meal to limit nausea while trametinib was administered ≥2 h after a meal as per the prescription information. Patients remained on study medications until disease progression, adverse events (AE) warranting discontinuation, or withdrawal of consent.

Once each dose combination had been cleared for safety, two to three additional subjects were enrolled in a trailing pharmacodynamic cohort. Mandatory pre- and post-treatment biopsies were obtained from these subjects. The planned Part 2 expansion cohort study was cancelled after review of results from Part 1.

### Patient selection

Subjects were eligible for the study if they were aged ≥18 years, had mesothelioma or solid tumours with a high likelihood of MAPK pathway activation, and had received ≥1 prior course of chemotherapy. Other key eligibility criteria included Eastern Cooperative Oncology Group (ECOG) performance status 0–1; adequate haematologic, hepatic, and renal function; ability to swallow oral medication; and left ventricular ejection fraction (LVEF) at or above the lower limit of normal. Female patients with childbearing potential were asked to comply with protocol-specified contraceptive measures, and male patients with female partners of childbearing potential needed to have had a prior vasectomy or agree to use effective contraception. Patients with symptomatic leptomeningeal or brain metastases were excluded, as were patients with active interstitial lung disease or pneumonitis; a history of retinal vein occlusion or central serous retinopathy; current use of warfarin; history or evidence of cardiovascular risk; and presence of active gastrointestinal disease or other condition that may interfere with the pharmacokinetics of the drugs.

### Dose-limiting toxicity

AEs were assessed throughout the study using the National Cancer Institute Common Terminology Criteria for Adverse Events v4.0 guidelines.^13^ An AE was considered a dose-limiting toxicity (DLT) if it had a causal or possible causal relationship to study treatment within the first 28 days and met the following criteria: ≥Grade 3 non-haematologic toxicity not controlled by routine supportive measures; Grade 4 neutropenia lasting >5 days; febrile neutropenia of any grade; Grade 4 thrombocytopenia; alanine aminotransferase >3x upper limit of normal (ULN) with bilirubin >2x ULN; any toxicity that resulted in missing ≥14 consecutive or non-consecutive days of scheduled dosing during the initial 28-day period; any Grade 2 toxicity that, in the investigator’s judgement, would be dose limiting.

### Pharmacokinetic analysis

Blood samples for pharmacokinetic analysis were collected prior to morning dosing of GSK2256098 on Day 15, and at 1, 1.5, 2, 4, 6, 8 h after the morning dose of GSK2256098. Trametinib was administered immediately after the 1.5-hour pharmacokinetic sample was collected.

For PK analysis of trametinib, blood samples were taken via an indwelling cannula (or by direct venepuncture), collected into a di-potassium ethylenediaminetetraacetic acid (K2-EDTA) tube and immediately placed on water ice. Blood samples stored on wet ice were centrifuged within 1 h of collection at approximately 1500 g for 10 min at 4 °C. Supernatant plasma was transferred to a 3.6 mL Nunc tube and stored at 20 °C before shipment. Samples were shipped frozen on dry ice at agreed timepoints throughout the study to GSK Pharmaceuticals, Upper Merion, PA, USA, for analysis.

For pharmacokinetic analysis of GSK2256098, blood samples were taken via an indwelling cannula (or by direct venepuncture), collected into a K2-EDTA tube and gently mixed by inversion of the capped tube 3 times until all the EDTA had dissolved into the sample. Approximately 15 μL blood was then transferred from the “end-to-end” EDTA capillary tube onto the Whatman FTA DBS card.

Plasma samples were analysed for trametinib using the validated analytical method based on liquid-liquid extraction, followed by high-performance liquid chromatography mass spectrometry (HPLC-MS/MS) analysis using a TurboIonSpray interface and multiple reaction monitoring. The lower limit of quantification (LLQ) for trametinib was 0.25 ng/mL using a 50-mL aliquot of human plasma with a higher limit of quantification (HLQ) of 250 ng/mL. Blood samples were analysed for GSK2256098 using a validated analytical method based on acetonitrile extraction from dried blood spots on the Whatman FTA DBS card, followed by HPLC/MS/MS analysis using a TurboIonSpray interface and multiple reaction monitoring. The LLQ for GSK2256098 was 10 ng/mL using a 3-mm diameter.

GSK2256098 in human blood was also analysed from blood/water (50/50, v/v) using the validated analytical method based on protein precipitation followed by HPLC-MS/MS analysis using a TurboIonSpray interface and multiple reaction monitoring. The LLQ for GSK2256098 was 10 ng/mL using a 25-μL aliquot of blood/water (50/50, v/v) with an HLQ of 10 000 ng/mL.

GSK2256098 whole-blood and dry blood spot concentration-time data as well as trametinib plasma concentration-time data were analysed by standard noncompartmental methods using Phoenix WinNonlin version 6.3. Calculations of all Pharmacokinetic parameters were based on doses of GSK2256098 and trametinib administered and actual sampling times from the respective dosing.

### Clinical activity

Clinical activity was assessed at baseline and every 8 weeks using the Response Evaluation Criteria In Solid Tumours (RECIST) 1.1 guidelines;^14^ mesothelioma was assessed using Modified RECIST for Mesothelioma.^15^

### Patient monitoring

Haematology and clinical chemistry laboratory tests were performed at baseline, Days 1, 8, 15, 22, 29, and every 4 weeks thereafter. Electrocardiograms were performed at baseline, Days 1, 15, 22, and then every 8 weeks from Day 1.

### Tumour biopsy collection and determination of pFAK levels

Paired tumour biopsies were collected prior to dosing on Day 1 and on a day from Days 15 to 22 inclusive. Samples were analysed for pFAK and total FAK by a proprietary Collaborative-Enzyme Enhanced Reactive-immunoassay (CEER) (Prometheus Laboratories, California, USA). A proximity-based immunoassay, the CEER assay leverages a multiplexed immune-microarray platform combined with triple-antibody-enzyme channelling signal amplification. Specific signal amplification occurs when target proteins (pFAK and FAK) captured on an antibody microarray co-localize with two additional detector-antibodies linked with channelling enzymes (horseradish peroxidase, HRP, and glucose oxidase, GO). pFAK levels were normalised to total FAK. The same technology was leveraged to analyse pERK and other MAPK pathway markers. However, a channel enzyme−enhanced reaction assay for total ERK could not be developed, so pERK was normalized to total FAK.

### Determination of Merlin status

Paraffin-embedded, archival tumour samples were required for all subjects. Merlin (the protein product of the *NF2* gene) status was determined by immunohistochemistry of formalin fixed paraffin-embedded (FFPE) archival samples collected from subjects with mesothelioma (*n* = 21). The immunohistochemistry assay was developed and conducted by Mosaic Laboratories (Lake Forest, California, USA). The primary antibody included an *NF2* antibody (C-18): sc332, Santa Cruz Biotechnology (Dallas, Texas, USA). A cell line tissue microarray comprising a total of six high, moderate, and low Merlin expressors and a rabbit IgG served as the positive and negative control, respectively.

Merlin status from archived tumour tissue was recorded as the percentage of cells that stain at + 2, + 3 using the above assay and then dichotomised as either “Merlin Negative,” if the percentage of neoplastic cells stained in the intensity staining category 3 and 2 equalled 0 or was less than 10 in category 2, or “Merlin Positive” otherwise.

### Statistical analysis

No formal statistical hypotheses were tested. An exploratory analysis of PFS was conducted for the subgroup of subjects with mesothelioma. PFS was defined as the time from the first dose of study drug to the first documented disease progression on radiological or clinical assessment, or to death from any cause. PFS was censored at the time of last radiological disease assessment in subjects who did not progress or die, and at the date of the first dose of study drug in subjects who discontinued the study with no post-treatment tumour assessment. Kaplan–Meier curves were produced for all mesothelioma subjects together and separately by Merlin status.

## Results

### Demographics and subject disposition

Thirty-four subjects were enrolled and received at least one dose of combination treatment. Of eight subjects who discontinued the study, seven withdrew consent and one was removed at the investigator’s discretion. Thirty-two subjects were included in the pharmacokinetic population and 21 in the pharmacodynamic cohort. Patient characteristics are shown in Supplementary Table 1. The mean ( ± SD) age of subjects was 64.4 years ( ± 9.95); 22 subjects (65%) were aged ≥65 years. Most subjects (*n* = 33; 97%) were white. Mesothelioma was the most common tumour type (*n* = 21; 62%).

### Determination of the MTD

Table 1 summarises doses and DLTs. Two of three subjects receiving trametinib 1 mg QD/GSK2256098 500 mg BID developed DLTs. As a result, the trametinib dose was reduced to 0.5 mg QD for Cohort 2, with GSK2256098 maintained at 500 mg BID. Of seven subjects enrolled, six were evaluable, and DLTs developed in two of these six subjects. Five subjects in Cohort 3 received trametinib 0.5 mg QD/GSK2256098 250 mg BID; one subject developed a DLT. Five additional subjects were enrolled at this dose for pharmacodynamic assessments. In Cohort 4, four subjects received trametinib 0.25 mg QD/GSK2256098 500 mg BID. Three subjects were evaluable, but none developed DLTs. Four additional subjects were enrolled at this dose for pharmacodynamic assessments. Cohort 5 had four subjects treated with trametinib 0.375 mg QD/GSK2256098 500 mg BID; one subject had a DLT. Two additional subjects were enrolled for pharmacodynamic assessment; there were no additional DLTs.

**Table 1:** Summary of dose-limiting toxicities

Cohort	Treatment	No. of subjects treated	No. of subjects with DLTs
1	GSK2256098 500 mg BID + trametinib 1.0 mg QD	3	2 – Grade 3 rash (pustular) - Grade 3 rash (dermatitis acneiform)
2	GSK2256098 500 mg BID + trametinib 0.5 mg QD	7	2 – Grade 3 decreased appetite - Grade 3 fatigue, Grade 3 rash (maculopapular)
3	GSK2256098 250 mg BID + trametinib 0.5 mg QD	10	1 – Grade 3 ejection fraction decreased
4	GSK2256098 500 mg BID + trametinib 0.25 mg QD	8	0
5	GSK2256098 500 mg BID + trametinib 0.375 mg QD	6	1 – Grade 2 dermatitis (acneiform)

*BID* twice daily, *DLT* dose-limiting toxicity, *PD* pharmacodynamics, *PK* pharmacokinetics, *QD* once daily

The high GSK2256098/low trametinib MTD was determined to be 500 mg BID/0.375 mg QD and the low GSK2256098/high trametinib MTD as 250 mg BID/trametinib 0.5 mg QD.

### Safety

All subjects reported at least 1 AE. AEs occurring in ≥20% of all subjects were nausea (59%), diarrhoea (53%), decreased appetite (38%), pruritus, fatigue (both 29%), rash (26%), vomiting (24%), and asthenia, cough, or acneiform dermatitis (21% each) (Table 2 and Supplementary Table 2). There were no grade 4 AEs. Twenty subjects (59%) had ≥1 Grade 3 AE; diarrhoea (*n* = 3), asthenia (*n* = 3), decreased appetite (*n* = 2), and fatigue (*n* = 2) were most common.

**Table 2:** Adverse events occurring in ≥10% of patients, regardless of causality

Adverse event	GSK2256098 250 mg BID + 0.5 mg trametinib QD (N = 10)	GSK2256098 500 mg BID + 0.25 mg trametinib QD (N = 8)	GSK2256098 500 mg BID + 0.375 mg trametinib QD (N = 6)	GSK2256098 500 mg BID + 0.5 mg trametinib QD (N = 7)	GSK2256098 500 mg BID + 1.0 mg trametinib QD (N = 3)	Total (N = 34)
Any event, n (%)	10 (100)	8 (100)	6 (100)	7 (100)	3 (100)	34 (100)
Nausea	5 (50)	4 (50)	3 (50)	5 (71)	3 (100)	20 (59)
Diarrhoea	5 (50)	3 (38)	4 (67)	3 (43)	3 (100)	18 (53)
Decreased appetite	4 (40)	1 (13)	2 (33)	5 (71)	1 (33)	13 (38)
Fatigue	2 (20)	3 (38)	1 (17)	2 (29)	2 (67)	10 (29)
Pruritus	3 (30)	3 (38)	0	1 (14)	3 (100)	10 (29)
Rash	3 (30)	2 (25)	0	2 (29)	2 (67)	9 (26)
Vomiting	1 (10)	1 (13)	1 (17)	4 (57)	1 (33)	8 (24)
Asthenia	2 (20)	0	3 (50)	2 (29)	0	7 (21)
Cough	2 (20)	2 (25)	2 (33)	1 (14)	0	7 (21)
Dermatitis acneiform	1 (10)	3 (38)	2 (33)	0	1 (33)	7 (21)
Constipation	3 (30)	2 (25)	0	0	0	5 (15)
Dyspnoea	1 (10)	2 (25)	1 (17)	1 (14)	0	5 (15)
Folliculitis	3 (30)	0	2 (33)	0	0	5 (15)
Anaemia	2 (20)	0	0	2 (29)	0	4 (12)
Gastro-oesophageal reflux disease	1 (10)	1 (13)	0	2 (29)	0	4 (12)
Oedema peripheral	1 (10)	1 (13)	1 (17)	0	1 (33)	4 (12)

Grade 3 adverse events occurring in three subjects: diarrhoea, asthenia; Grade 3 adverse events occurring in two subjects: decreased appetite, fatigue; Grade 3 adverse events occurring in one subject: pruritus, dermatitis acneiform, constipation, dyspnoea, anaemia, increased serum creatinine, lower respiratory tract infection, maculopapular rash, atrial fibrillation, confusion, pulmonary embolism, acute myocardial infarction, cancer pain, dehydration, decreased ejection fraction, bone metastases, neutropenia, pericardial effusion, pneumonitis, pneumothorax, pustular rash.

*BID* twice daily, *QD* once daily

Fourteen subjects developed serious AEs, most commonly diarrhoea (*n* = 2), lower respiratory tract infection (*n* = 2), nausea (*n* = 2), and vomiting (*n* = 2), although none of these were considered related to GSK2256098; 15% of subjects had an SAE that could be attributed to trametinib. Sixteen subjects (47%) had ≥1 AE leading to dose interruption. Seventeen percent of subjects in the group receiving high GSK2256098/low trametinib (500 mg BID/0.375 mg QD) had an AE causing dose interruption compared with 60% in the low GSK2256098/high trametinib (250 mg BID/0.5 mg QD) group. Five subjects had a dose reduction due to non-serious AEs, with no notable differences across dose groups. The trametinib dose was reduced in three subjects, the GSK2256098 dose in one, and the dose of both agents in another.

### Pharmacokinetics and pharmacodynamics

Peak plasma concentration (C_max_) and area under the concentration-time curve over the dosing interval (AUC_(0-τ)_) were generally dose proportional for both agents (Table 3). Trametinib exposure (*C*_max_ and AUC) was 2- to 4-fold higher when administered with GSK2256098 versus trametinib monotherapy data (Table 3). The exposure of GSK2256098 was not altered when co-administered with trametinib. The median time to *C*_max_ (*t*_max_) for GSK2256098 was generally 2 h post-dose for GSK2256098 and 1.5 to 2.5 h post-dose for trametinib. The *t*_max_ of trametinib was slightly delayed with GSK2256098 co-administration.

**Table 3:** GSK2256098 and trametinib pharmacokinetic parameters after repeat dosing on Day 15

	C_max_, ng/mL Geometric mean (%CV)	t_max_, h Median (range)	AUC_(0–τ)_, ng·h/mL Geometric mean (%CV)
*GSK2256098 pharmacokinetics when given in combination with trametinib (whole blood)*			
GSK2256098 250 mg (*n* = 10)	1397 (71)	2.0 (1.5–4.1)	5467 (58)
GSK2256098 500 mg (*n* = 21)	2453 (82)	2.0 (1.5–6.0)	10 410^a^ (62)
*GSK2256098 pharmacokinetics when given in combination with trametinib (dry blood spot)*			
GSK2256098 250 mg (*n* = 6)	1229 (73)	2.8 (1.5–4.1)	4975 (57)
GSK2256098 500 mg (*n* = 10)	2834 (44)	2.0 (1.5–4.0)	12 663 (27)
*Trametinib pharmacokinetics when given in combination with GSK2256098 (plasma)*			
Trametinib 0.25 mg + GSK2256098 500 mg BID (*n* = 6)	6.23 (17)	2.5 (0.33–6.5)	122 (15)
Trametinib 0.375 mg + GSK2256098 500 mg BID (*n* = 6)	11.2 (47)	2.5 (2.0–6.0)	229 (43)
Trametinib 0.5 mg + GSK2256098 250 mg BID (*n* = 10)	16.3 (35)	2.4 (0.50–6.5)	347^b^ (33)
Trametinib 0.5 mg + GSK2256098 500 mg BID (*n* = 6)	13.0 (27)	2.6 (0.62–4.8)	266 (26)
Trametinib 1.0 mg + GSK2256098 500 mg BID (*n* = 3)	36.0 (11)	1.5 (1.5–6.5)	732 (22)
*Trametinib pharmacokinetics when given as monotherapy (plasma)* ^16^			
Trametinib 2.0 mg (*n* = 13)	22.2 (28)	1.75 (1.0–3.0)	370^c^ (22)

*AUC* area under the concentration-time curve over the dosing interval, *BID* twice daily, *C*_max_, maximal plasma concentration, *QD* once daily, *t*_max_ time to *C*_max_, *% CV* between-subject coefficient of variation

^a^*n* = 20

^b^*n* = 8

^c^AUC in this study was reported as 0–24 h

There was good agreement between dried blood spot and whole blood concentrations of GSK2256098 (Table 3).

**Fig. 1:**
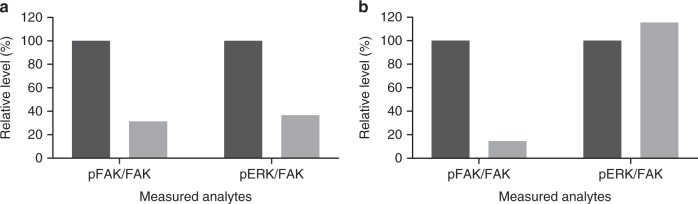
Pharmacodynamic response in tumour samples evaluated at baseline (pre-dose) and kpost-dosing of GSK2256098 and trametinib for 22 days. The expression level relative to baseline levels (normalized to 100%; Dark grey bars) of pFAK and pERK normalised to the level of FAK in the sample. The ratio of the pFAK level relative to FAK and the pERK level relative to FAK are shown for each subject and are indicative of kinase inhibition by GSK2256098 and trametinib, respectively (Light grey bars). **a**) Subject #200 dosed with GSK2256098 at 500 mg BID and trametinib at 0.5 mg QD. **b** Subject #202 dosed with GSK2256098 at 250 mg BID and trametinib at 0.5 mg QD

Paired biopsies were available for eight subjects and phosphorylated FAK (pFAK)/FAK was determined for six; two subjects with insufficient post-dose tumour tissue were not evaluable. Three of six subjects showed >70% decrease in pFAK/FAK from baseline; two showed >45% reduction in pFAK/FAK levels. The remaining subject demonstrated an increased pFAK/FAK ratio (Table 4). pERK data were obtained in only three of the eight subjects showing a divergent trend: two subjects had >70% inhibition of pFAK and >60% inhibition of pERK (Fig. 1a; example data for subject #200); the other had activation of pERK (Fig. 1b; example data for subject #202). The remaining five subjects had signals below the lower limit of quantification. Merlin status was determined for 18/21 archival tumour samples from subjects with mesothelioma; 15 (71%) samples stained negative for Merlin.

**Table 4:** Pharmacodynamic response for pFAK in pre- and post-dose tumour samples

Subject No.	Visit	pFAK (CU/μg)	Total FAK (CU/μg)	pFAK/FAK	% Change from baseline pFAK	% Change from baseline pFAK/FAK
*GSK2256098 250* mg *BID* + *trametinib 0.5* *mg QD*						
509	Baseline	112.070	1329.1	0.084	NA	NA
	Day 15	13.950	585.9	0.024	–87.6	–71.763
510	Baseline	92.400	103.3	0.895	NA	NA
	Day 15	60.860	245.7	0.248	–34.1	–72.310
202	Baseline	189.010	680.3	0.278	NA	NA
	Day 22	30.960	762.1	0.041	–83.6	–85.378
206	Baseline	99.580	207.3	0.480	NA	NA
	Day 22	50.110	59.2	0.846	–49.7	76.125
*GSK2256098 500* *mg BID* *+* *trametinib 0.25* *mg QD*						
507	Baseline	72.750	1710.6	0.043	NA	NA
	Day 15	NA	1107.3	NA	NA	NA
121	Baseline	41.680	183.7	0.227	NA	NA
	Day 22	25.900	214.6	0.121	–37.9	–46.809
*GSK2256098 500* *mg BID* *+* *trametinib 0.375* *mg QD*						
118	Baseline	79.470	268.6	0.296	NA	NA
	Day 22	NA	NA	NA	NA	NA
*GSK2256098 500* *mg BID* + *trametinib 0.5* *mg QD*						
200	Baseline	15.230	12.1	1.263	NA	NA
	Day 22	24.600	62.0	0.397	−61.5	–68.586

*BID* twice daily, *FAK* focal adhesion kinase, *NA* not available, *pFAK* phosphorylated FAK, *QD* once daily

### Clinical activity

**Fig. 2:**
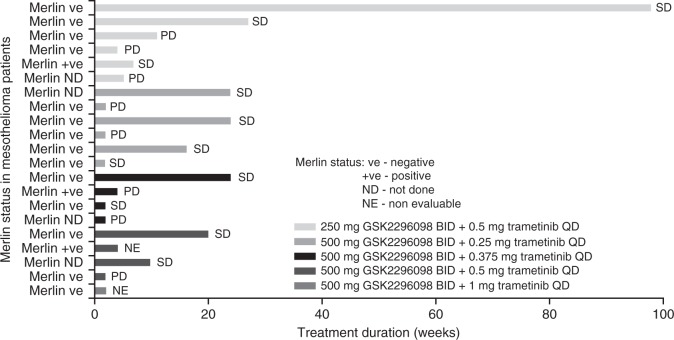
Plot of duration on treatment and best response in subjects with mesothelioma. BID twice daily, PD progressive disease, QD once daily, SD stable disease

No objective responses to treatment were reported. The best response was stable disease in 13 subjects (38%). Median PFS was 11.8 weeks (95% CI: 6.1, 24.1) for subjects with mesothelioma. Median PFS for 14 mesothelioma subjects with Merlin-negative tumours was 15.0 weeks (95% CI: 4.4, 24.1) and 7.3 weeks for 3 subjects with Merlin-positive tumours (CI not calculable) (Fig. 2).

## Discussion

This is the first clinical study evaluating the combination of FAK and MEK inhibitors in subjects with cancer. Preclinical studies of the combination demonstrated synergistic activity in several tumour types, including mesothelioma, providing justification for evaluating this combination.

We used an adaptive design to allow simultaneous evaluation of multiple dose levels, starting at 50% of the MTD of GSK2256098 and 50% of the recommended dose of trametinib.^7,12^ DLTs at the first dose level required dose de-escalation. Initially we assumed that the excessive toxicity resulted from overlapping effects on signal transduction pathways. However, pharmacokinetic analysis showed that systemic exposure of trametinib was unexpectedly high when co-administered with GSK2256098. After dose normalization, the geometric mean C_max_ and AUC for trametinib were 2- to 4-fold higher when trametinib was administered concomitantly with GSK2256098 compared with trametinib monotherapy.^16^ Our data also suggest that t_max_ may be slightly delayed when trametinib is co-administered with GSK2256098 relative to the monotherapy t_max_.^16^ The pharmacokinetics of GSK2256098 were unaffected by concomitant trametinib and were comparable to those seen in a previous monotherapy study.^7^

Trametinib is metabolised predominantly via deacetylation (non-CYP mediated) alone or with mono-oxygenation or in combination with glucuronidation biotransformation pathways. Although the enzyme responsible is unidentified, deacetylation is likely mediated by hydrolytic esterases, such as carboxylesterases or amidases. The mechanism of the pharmacokinetic interaction between GSK2256098 and trametinib is not fully understood, but *in vitro* investigation found that GSK2256098 inhibits deacetylation of trametinib by inhibiting human carboxylesterases (eg, hCES1b, hCES1c, and hCES2) and potentially other esterases (GSK data on file). The increased trametinib plasma levels may result from inhibition of its metabolic pathway. GSK2256098 also appears to impact trametinib’s absorption, based on the delayed t_max_ in this study relative to historical trametinib monotherapy data.^16^

Several strategies are used to determine doses of anticancer agents administered in combination. Although it is tempting to use the MTD of the more active agent and titrate up the investigational agent until the MTD is identified, such an approach in the current study could have resulted in life-threatening AEs. Fortunately, by adjusting down the dose of trametinib, MTDs for both low trametinib/high GSK2256098 and high trametinib/low GSK2256098 combinations were determined. The clinical concentrations at these doses are within the range of those associated with the *in vitro* gIC_50_ and dEC_50_ for both compounds.^6,17^

Except at the initial dose levels, the overall safety of the combination was acceptable. Of note was the better tolerability of the high GSK2256098/low trametinib MTD versus the low GSK2256098/high trametinib dose level.

The results of the present study showed almost equivalent effect between GSK2256098 + trametinib combination and those from an earlier GSK2256098 monotherapy study^7^ in terms of reduction of FAK levels. The addition of trametinib did not show any clinically relevant benefit. Pharmacodynamic studies demonstrated that GSK2256098 inhibited pFAK at the doses evaluated. An earlier GSK2256098 monotherapy study similarly found that patients with Merlin-negative mesothelioma had longer PFS than those with Merlin-positive mesothelioma.^7^ The prognostic significance of Merlin status has been recently reported by Meerang.^18^ Meerang et al reported in two independent cohorts of mesothelioma patients that low tumour Merlin expression is associated with a poorer freedom from recurrence and poorer overall survival than in mesothelioma patients with higher Merlin expression. Despite low Merlin tumour expression being a negative prognostic factor in patients with mesothelioma, our results suggest that mesothelioma patients with absent or low Merlin expression have a longer duration of treatment than Merlin-positive patients when administered GSK2256098.^18^ These current findings confirm the prior monotherapy findings of GSK2256098.

A greater effect of FAK inhibition in Merlin-negative than Merlin-positive mesothelioma tumours is consistent with data showing that Merlin inactivation in mesothelioma cell lines is related to invasiveness and upregulation of the FAK pathway.^19^

In conclusion, we identified MTDs for a GSK2256098/trametinib combination for use in future clinical studies. There was, however, limited support for efficacy of this combination in these patients. We also highlight the importance of pharmacokinetic monitoring and careful selection of starting doses during phase Ib dose-escalation combination studies, even where no pharmacokinetic interaction between investigational agents is anticipated.

## Supplementary information

CL-2018-6313R_Supplementary Material

## Data Availability

Summaries of the study protocol and results are available from https://gsk-clinicalstudyregister.com/search/?study_ids=FAK114746. Anonymized individual participant data and study documents can be requested for further research from www.clinicalstudydatarequest.com.
