# Supplementary material for: A phase Ib dose-finding, pharmacokinetic study of the focal adhesion kinase inhibitor GSK2256098 and trametinib in patients with advanced solid tumours
Source: Br J Cancer. 2019 Apr 17;120(10):975–81. doi: 10.1038/s41416-019-0452-3 (PMC6735221; doi:10.1038/s41416-019-0452-3)
Supplement: Supplementary file 1 — CL-2018-6313R_Supplementary Material [file 41416_2019_452_MOESM1_ESM.docx]

# Supplementary Information

**Supplementary table 1. Patient characteristics**

| **Characteristic** | **Patients (*n =* 34)** |
| --- | --- |
| Age, years |  |
| Median (range) | 66 (30–78) |
| Mean (±SD) | 64.4 (9.95) |
| Gender, n (%) |  |
| Male | 18 (53) |
| Female | 16 (47) |
| ECOG performance status, n (%) |  |
| 0 | 15 (44) |
| 1 | 19 (56) |
| Race, n (%) |  |
| White | 32 (97) |
| White and Arabic/North African heritage | 1 (3) |
| Unknown | 1 (3) |
| Tumor types, n (%) |  |
| Mesothelioma | 21 (62) |
| Colorectal cancer | 3 (9) |
| Endometrial/uterine cancer | 2 (6) |
| Non-small-cell lung cancer | 2 (6) |
| Other* | 6 (18) |

*Esophageal cancer (*n* = 1), pancreatic cancer (*n* = 1), prostate cancer (*n* = 1), small intestinal cancer (*n* = 1), bronchial squamous cell carcinoma (*n* =1), unknown primary cancer (*n* = 1)

ECOG, Eastern Cooperative Oncology Group; SD, standard deviation

**Supplementary Table 2. Grade 3 adverse events categorized according to their frequency of incidence**

| n=3 | n=2 | n=1 |
| --- | --- | --- |
| Asthenia  Diarrhoea | Decreased appetite Fatigue | Acute myocardial infarction  Anemia  Atrial fibrillation  Bone metastases  Cancer pain  Confusion  Constipation  Decreased ejection fraction  Dehydration  Dermatitis acneiform  Dyspnoea  Increased serum creatinine  Infection  Lower respiratory tract  Maculopapular rash  Neutropenia  Pericardial effusion  pneumonitis,  Pneumothorax  Pruritus  Pulmonary embolism  Pustular rash |
